# XMR: an explainable multimodal neural network for drug response prediction

**DOI:** 10.3389/fbinf.2023.1164482

**Published:** 2023-08-02

**Authors:** Zihao Wang, Yun Zhou, Yu Zhang, Yu K. Mo, Yijie Wang

**Affiliations:** ^1^ Department of Computer Science, Indiana University Bloomington, Bloomington, IN, United States; ^2^ Department of Environmental and Occupational Health, School of Public Health, Indiana University Bloomington, Bloomington, IN, United States; ^3^ Department of Epidemiology and Biostatistics, School of Public Health, Indiana University Bloomington, Bloomington, IN, United States

**Keywords:** drug response prediction, machine learning, interpretable deep learning, multimodal deep learning, triple-negative breast cancer

## Abstract

**Introduction:** Existing large-scale preclinical cancer drug response databases provide us with a great opportunity to identify and predict potentially effective drugs to combat cancers. Deep learning models built on these databases have been developed and applied to tackle the cancer drug-response prediction task. Their prediction has been demonstrated to significantly outperform traditional machine learning methods. However, due to the “black box” characteristic, biologically faithful explanations are hardly derived from these deep learning models. Interpretable deep learning models that rely on visible neural networks (VNNs) have been proposed to provide biological justification for the predicted outcomes. However, their performance does not meet the expectation to be applied in clinical practice.

**Methods:** In this paper, we develop an XMR model, an eXplainable Multimodal neural network for drug Response prediction. XMR is a new compact multimodal neural network consisting of two sub-networks: a visible neural network for learning genomic features and a graph neural network (GNN) for learning drugs’ structural features. Both sub-networks are integrated into a multimodal fusion layer to model the drug response for the given gene mutations and the drug’s molecular structures. Furthermore, a pruning approach is applied to provide better interpretations of the XMR model. We use five pathway hierarchies (cell cycle, DNA repair, diseases, signal transduction, and metabolism), which are obtained from the Reactome Pathway Database, as the architecture of VNN for our XMR model to predict drug responses of triple negative breast cancer.

**Results:** We find that our model outperforms other state-of-the-art interpretable deep learning models in terms of predictive performance. In addition, our model can provide biological insights into explaining drug responses for triple-negative breast cancer.

**Discussion:** Overall, combining both VNN and GNN in a multimodal fusion layer, XMR captures key genomic and molecular features and offers reasonable interpretability in biology, thereby better predicting drug responses in cancer patients. Our model would also benefit personalized cancer therapy in the future.

## 1 Introduction

Precision medicine is a key challenge in this century, with a focus on personalized cancer treatments. Precision medicine aims to design treatments specific to a patient’s molecular profile, improving outcomes. This relies on effectively using clinical, genomics, and other “omics” data to identify prognostic and predictive biomarkers. Another important task for precision oncology is to generate drug response profiles across drugs and cancer subtypes. Large-scale drug screening initiatives (Barretina et al., 2012; Yang et al., 2012; Basu et al., 2013; Seashore-Ludlow et al., 2015) have made data publicly available, enabling the identification of biomarkers and the development of predictive models like elastic net and random forest (Iorio et al., 2016). However, the task of predicting drug response is complex due to the genetic heterogeneity among cancer patients, which presents a major obstacle in determining therapeutic efficacy (Bedard et al., 2013; Dagogo-Jack and Shaw, 2018; Fittall and Van Loo, 2019; Lim and Ma, 2019; Ramón y Cajal et al., 2020). Despite advances in the field, there is still a need for further improvement in the accuracy and reliability of drug response models. Deep learning (DL) is well suited for drug response prediction, as it can handle large amounts of high-dimensional data and capture non-linear relationships in biological data better than other machine learning algorithms. DL has been successful in a variety of drug discovery tasks and may outperform traditional machine learning approaches in drug response prediction (Yuan et al., 2016; Luo et al., 2019; Sun et al., 2019; Jiao et al., 2020) despite being underexplored until recently.

A challenge in drug response prediction is to accurately represent both the genotype and chemical structures of drugs. However, most studies have focused on enhancing genotype representation while neglecting the chemical side, resulting in models with strong genotypic embedders and weak chemical embedders (Kuenzi et al., 2020; Huang X. et al., 2021). However, this imbalance can negatively impact performance as the chemical structure of drugs contains valuable information that requires a stronger embedder, while the genotypic information is prone to overfitting and, thus, needs a lighter architecture for better generalizability. To address this issue, in this paper, we develop an XMR model, an eXplainable Multimodal neural network for drug Response prediction. Our approach emphasizes the importance of having a powerful chemical embedder while keeping the genotypic embedder relatively lightweight. To achieve this, XMR is structured as a multimodal neural network with two sub-networks: a visible neural network (VNN) for capturing genomic features and a graph neural network (GNN) for learning the structural features of drugs. To enhance the generalizability of genotypic embedding, the VNN is further pruned to form a more compact structure.

In this study, we used XMR to construct cancer-specific models for triple-negative breast cancer (TNBC) to gain a deeper insight into its biological mechanisms. The XMR models were built based on five key pathways (cell cycle, DNA repair, diseases, signaling transduction, and metabolism). These models were trained on TNBC-specific data obtained from the Cancer Therapeutics Response Portal (CTRP) v2 (Seashore-Ludlow et al., 2015) and the Genomics of Drug Sensitivity in Cancer (GDSC) database (Yang et al., 2012). To demonstrate the effectiveness of XMR’s models, we compared their predictive accuracy to several state-of-the-art methods using validation samples. Our results showed that XMR outperforms these methods with significantly higher test accuracy. In addition, we evaluated the explainability of XMR. We found that our model was able to capture the commonly mutated TNBC-related genes, several critical pathways (e.g., G2/M checkpoint, PI3K/mTOR, and MAPK pathways), and novel drugs that would provide insights into TNBC treatment (e.g., dinaciclib, panobinostat, BI 2536, and AZD7762).

## 2 Materials and methods

### 2.1 Taxonomy of multimodal models

In this paper, we formulate the drug response prediction task as a multimodal learning task, utilizing two forms of information: the genotype represented by binary mutations and the chemical structure of drugs. The current research focusing on multimodal models is primarily developing models that can effectively combine and process information from multiple modalities such as audio, text, images, and video. We believe that the insights and results from developing multimodal models for vision and language tasks can be effectively applied to the task of predicting drug response.

**FIGURE 1:**
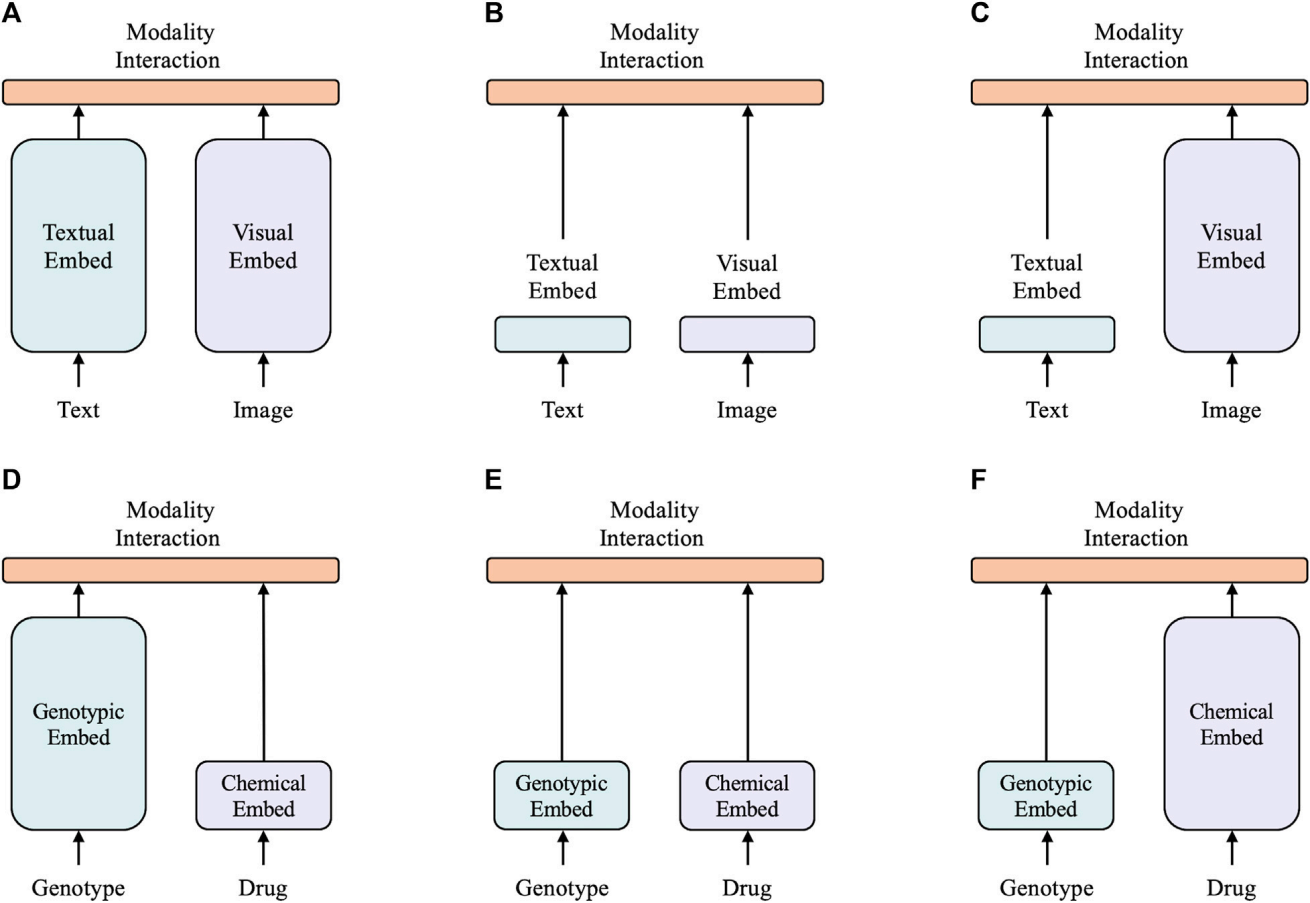
Six categories of multimodal models, with the height of each rectangle indicating its comparative computational size. **(A)** Twin tower model in vision-and-language domain. **(B)** Shallow model in vision-and-language domain. **(C)** Vision-and-language model with heavy textual embedder. **(D)** Model with heavy genotype embedder. **(E)** Shallow model in drug response prediction domain. **(F)** Model with heavy chemical embedder.

A taxonomy of multimodal models is proposed based on two factors: 1) the task, which can be vision and language tasks, or the drug response prediction task, and 2) the expressiveness level of the two modalities in terms of dedicated parameters or computation. This results in four archetypes as shown in Figure 1.

The top three archetypes are vision-and-language models. CLIP (Radford et al., 2021) is a typical twin tower model, as shown in Figure 1A, as it employs separate but similarly expensive embedders for each modality. Despite CLIP’s remarkable zero-shot performance in image-to-text retrieval, its performance was not as strong as other vision-and-language downstream tasks. ViLT (Kim et al., 2021) is a (Figures 1A, B) shallower and computationally lighter model with shallow embedding layers for raw pixels and text tokens. Most computations of ViLT focus on modeling modality interactions. This simple architecture provides faster inference time, but it has a slow training process due to its light visual embedder. Its performance is also limited in many tasks. Most state-of-the-art models (Lu et al., 2019; Chen et al., 2020; Li et al., 2021) belong to the archetype shown in Figure 1C, with a visual embedder much heavier than the textual embedder. This type of model generally achieves the highest performance in various vision-and-language tasks. This demonstrates that most vision-and-language tasks necessitate a powerful visual feature extractor, i.e., a heavier visual embedder, with the textual embedder being relatively lightweight. Intuitively, in the field of drug response prediction, the features of drugs can be compared to visual features as the chemical structure holds rich information, similar to images. On the other hand, genotypic features can be related to textual features as they are both binary and discrete. Hence, our hypothesis is that a successful model for drug response prediction should have a heavy chemical embedder with a relatively light genotypic embedder.

The bottom three archetypes are for drug response prediction. DrugCell (Kuenzi et al., 2020) falls under the archetype shown in Figure 1D and utilizes a deep visible neural network to extract features from genotypes and a simple multilayer perceptron (MLP) to extract features from the Morgan fingerprint of drugs. The VNN maps the neurons of a deep neural network into potential molecular components and pathways in a biological structure, which is commonly used in various cancer studies (Ma et al., 2018; Wang et al., 2018; Kuenzi et al., 2020; Elmarakeby et al., 2021). However, it is often deep and substantial due to its utilization of biological networks. ParsVNN (Huang X. et al., 2021), which falls under the archetype shown in Figure 1E, improves upon DrugCell by using a sparse learning approach to learn a simplified VNN that only contains biological architectures most relevant to the prediction task. This results in ParsVNN having a better performance than DrugCell, which confirms our hypothesis of having a lighter genotypic embedder in drug response prediction models. Our proposed XMR, belonging to the archetype shown in Figure 1F, is the first model of its kind. It follows our hypothesis that a successful model should have a lightweight genotypic embedder and a relatively heavy chemical embedder. To implement this, XMR uses a deep graph neural network to extract more complex information from the chemical structure while following a design similar to ParsVNN for the genotypic embedder.

### 2.2 Model architecture

**FIGURE 2:**
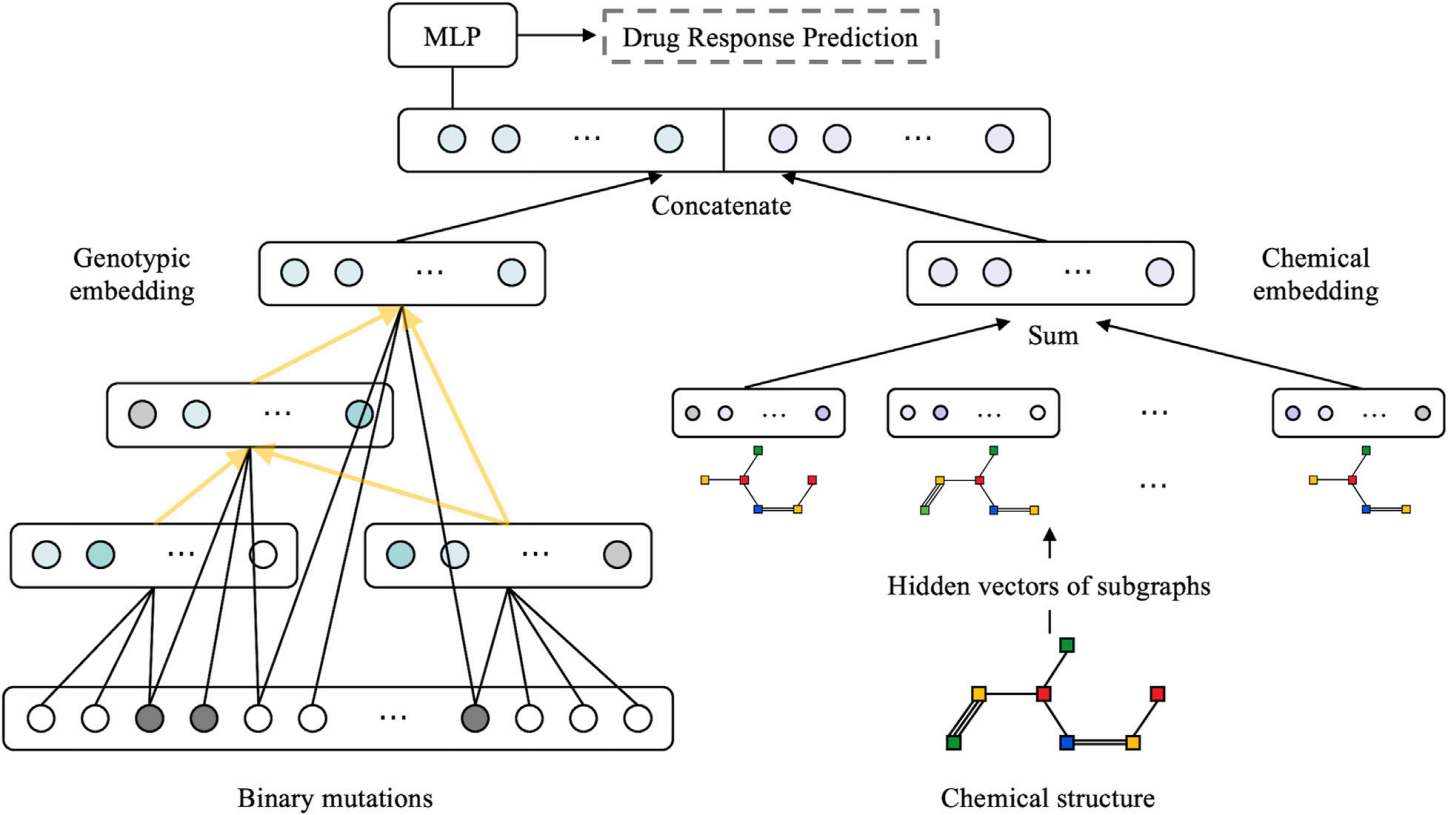
Overview of the proposed XMR architecture. A combination of genotypic and chemical embeddings, produced by a VNN (left) and a GNN (right), is concatenated and fed into a MLP layer for drug response prediction. The VNN architecture is represented by black arrows linking genes and yellow arrows representing molecular subsystems.

The model is structured as a multimodal neural network with two sub-networks: a visible neural network to capture genomic features and a graph neural network to learn the structural features of drugs (as illustrated in Figure 2). We followed the method described by Kuenzi et al. (2020) to build VNN embedding. Briefly, the VNN model establishes a connection between gene-level data and their associated phenotypic response in a cell. The VNN architecture resembles the hierarchical structure of cellular molecular subsystems, where artificial neurons represent molecular events and edges represent the connectivity among a series of related molecular events. The hierarchical structure of the VNN was created using pathways related to the cell cycle, DNA repair, diseases, signal transduction, and metabolism, respectively, as documented in the Reactome database (Fabregat et al., 2018). Each term in the pathway is represented by a hidden layer, and the hidden layers are interconnected precisely according to the molecular subsystems.

In more detail, the embedding for each term *ν*_*i*_ is composed of gene neurons, $\nu_{i}^{\mathrm{gene}}=\left\{\nu_{1}^{\mathrm{gene}},\ldots ,\nu_{p}^{\mathrm{gene}}\right\}$ , which take the genes directly connected to this term as an input, and subsystem neurons, $\nu_{i}^{\mathrm{sub}}=\left\{\nu_{1}^{\mathrm{sub}},\ldots ,\nu_{q}^{\mathrm{sub}}\right\}$ , which take the outputs of its child terms as an input. That is, $\nu_{i}=\left[\nu_{i}^{\mathrm{gene}}:\nu_{i}^{\mathrm{sub}}\right]$ . Then, the final genotypic embedding is represented by the embedding of the root term, i.e., **y**_*genotype*_ = *ν*_*root*_.

To learn chemical embedding, we used the GNN, which regards each atom of a compound as a node. The atoms can exchange information through their chemical bonds. The fundamental concept of the GNN is to iteratively gather information from the neighbors of each node (i.e., atom) so that each individual atom is aware of the molecular substructures surrounding it. To address the challenges of limited learning parameters and ineffective embedding learning due to the insufficient number of atom and bond types in the molecule, we followed the method described in Costa and De Grave (2010), which embeds compounds using *r* radius subgraphs, which are induced by neighboring vertices and edges within a radius of *r* from a vertex. In detail, a graph is represented as *G* = (*V*, *E*), where *V* is the set of vertices and *E* is the set of edges. In a molecule, *v*_*i*_ ∈ *V* represents the *i*th atom, and *e*_*ij*_ ∈ *E* represents the chemical bond between the *i*th and *j*th atoms. Given a graph *G* = (*V*, *E*), we represent a set of all neighboring vertex indices within a radius of *r* from the *i*th vertex as $v_{i}^{r}$ . Then, the *r*-radius subgraph for vertex *v*_*i*_ is defined as $G_{\mathrm{sub}}\left(v_{i},r\right)=\left(v_{i}^{r},e_{i}^{r}\right)$ , where $e_{i}^{r}=\left\{e_{mn}\in E|\left(m,n\right)\in v_{i}^{r}\times v_{i}^{r-1}\right\}$ . Additionally, the *r*-radius subgraph for edge *e*_*ij*_ is defined as $G_{\mathrm{sub}}\left(e_{ij},r\right)=\left(v_{i}^{r-1}\cup v_{j}^{r-1},e_{i}^{r}\cap e_{j}^{r}\right)$ . Each subgraph for the *r*-radius vertex and *r*-radius edge is then represented by a unique hidden vector.

Then, we describe the transition function for updating both the vertex and edge embeddings. Given a graph *G* and the initial embeddings of its vertices and edges, we represent the embedding of the *i*th vertex at time step *t* as $v_{i}^{\left(t\right)}$ . This embedding is updated using the following transition function:

$$v_{i}^{\left(t+1\right)}=Sigmoid\left(v_{i}^{\left(t\right)}+\Sigma_{j\in v_{i}^{r}}h_{ij}^{\left(t\right)}\right),$$ (1)

where the sigmoid function is defined as $Sigmoid\left(x\right)=\frac{1}{1+e^{-x}}$ , $v_{i}^{r}$ is the set of indices of neighboring vertices of *i*, and $h_{ij}^{\left(t\right)}$ is the hidden neighborhood vector. This hidden vector is calculated by considering the neighboring vertex *v*_*j*_ and edge *e*_*ij*_:

$$h_{ij}^{\left(t\right)}=ReLU\left(W\left[\begin{array}{c} v_{j}^{\left(t\right)}\\ e_{ij}^{\left(t\right)} \end{array}\right]+b\right),$$ (2)

where the ReLU function is defined as ReLU(*x*) = max(0, *x*), **W** is a weight matrix, **b** is a bias vector, and $e_{ij}^{\left(t\right)}$ is the edge embedding between the *i*th and *j*th vertices at time step *t*. By adding up the neighboring hidden vectors and iterating over time steps, the vertex embeddings can gradually accumulate more global information about the graph.

The procedure for updating edge embeddings is similar. Specifically, the edge embedding between the *i*th and *j*th vertices at time step *t*, $e_{ij}^{\left(t\right)}$ , is updated as follows:

$$e_{ij}^{\left(t+1\right)}=Sigmoid\left(e_{ij}^{\left(t\right)}+ReLU\left(W\left(v_{i}^{\left(t\right)}+v_{j}^{\left(t\right)}\right)+b\right)\right).$$ (3)

Thus, the final chemical embedding is obtained by taking the average of the vertex vectors obtained through the transition function, given the set $V=\left\{v_{1}^{\left(t\right)},v_{2}^{\left(t\right)},\ldots ,v_{n}^{\left(t\right)}\right\}$ , where *n* is the number of vertices in the molecular graph:

$$y_{\mathrm{drug}}=\frac{1}{n}\Sigma_{i=1}^{n}v_{i}^{\left(t\right)}.$$ (4)

We then combine **y**_*genotype*_ and **y**_*drug*_ into a single vector, [**y**_*genotype*_: **y**_*drug*_], and input it into a multilayer perceptron to make the final prediction of the drug response.

To create a more compact genotypic embedder, we follow the approach described by Huang X. et al. (2021). This method aims to simplify the VNN architecture while retaining its ability to make accurate predictions. It is based on the idea that biological processes are complex and involve many components and that sparse coding can capture the most significant components that are more directly relevant to drug administration and treatment, compared to considering all potential processes. Starting from a VNN model, we treat each edge weight as a feature of the VNN and perform sparse learning to improve prediction accuracy and select important features. This helps eliminate redundant features and improve the explainability of the downstream analysis. To accomplish this, we utilize *ℓ*_0_ norm regularization to prune edges between genes and subsystems and group LASSO regularization to remove edges between subsystems. The optimization problem is solved using the proximal alternating linearized minimization (PALM) algorithm (Bolte et al., 2014).

### 2.3 Explainability in XMR

When evaluating deep learning models, it is crucial to consider not only their prediction performance but also their ability to provide explanations. Explanations can come in two forms: global and local (Du et al., 2020). Global explanations offer a comprehensive understanding of how the model operates by examining its structure and parameters. Local explanations, on the other hand, focus on explaining why a specific prediction was made by the model by analyzing the causal relationship between the input and the prediction. Both types of explanations serve important purposes. Global explanations increase the transparency of deep learning models, while local explanations build trust in individual predictions. The XMR model focuses on global explainability, as it filters out the important pathways and genes that contribute the most to the prediction task for each cancer type and biological network. This provides insights into how XMR operates and offers guidance for building models for specific cancer types. Additionally, the model’s ability to predict drug response can be used to identify new drugs that may have a significant impact on a particular cancer type. We delve into the explainability provided by XMR in a later section. It is important to note that the explainability of the XMR model is not solely dependent on the VNN architecture but also on the overall architecture, since the model is trained end-to-end. The quality of the explainability is directly proportional to the model’s performance. The better the model, the more meaningful the guidance it can provide.

### 2.4 Dataset and splitting

We obtained the drug response data from GDSC (Yang et al., 2012) and the CTRP (Seashore-Ludlow et al., 2015). TNBC cell lines were selected according to the cell lines listed in Chavez et al. (2010) and Dai et al. (2017). A total of 22 TNBC cell lines were selected: BT20, BT549, CAL120, CAL148, CAL51, CAL851, DU4475, HCC1143, HCC1187, HCC1395, HCC1599, HCC1806, HCC1937, HCC2157, HCC38, HCC70, HDQP1, MDAMB157, MDAMB231, MDAMB436, MDAMB468, and MFM223. Those cell lines covered all the TNBC subtypes as described in Lehmann et al. (2011), including two basal-like (BL1 and BL2), an immunomodulatory (IM), a mesenchymal (M), a mesenchymal stem-like (MSL), and a luminal androgen receptor (LAR). The mutation status was collected from the DepMap portal (DepMap, 2022). A gene was selected if at least one of the chosen cell lines had a mutation on it. A total of 6,982 genes were identified. The mutation status of the gene was recorded as a binary variable and was either “1” for mutated or “0” for non-mutated. This procedure yielded 4,851 (cell line and drug) pairs for the final data, including 22 cell lines and 279 drugs. It was split into training and validation sets in an 8:2 proportion, resulting in 3,880 training samples and 971 validation samples. A separate test set was formed using all the (cell line and drug) pairs that were not present in the training and validation sets.

### 2.5 Construction of TNBC-specific XMR models

The VNN architecture in XMR was built based on five key biological networks (cell cycle, DNA repair, diseases, signaling transduction, and metabolism). Each term in the biological networks used in VNNs is comprised of a hidden layer with three neurons, while in the GNN, subgraphs with a radius of 2 are formed and represented by a hidden layer with 256 neurons. The drug response is measured using the area under the dose–response curve (AUC), where a lower AUC value indicates a more effective drug response, and normalization is carried out such that AUC = 0 represents complete cell death and AUC = 1 represents no effect. The prediction accuracy of XMR was evaluated using Spearman’s correlation between predicted and observed AUC values. The model was trained for 300 epochs with a batch size of 200, and a mean squared error loss was used, with an AdamW optimizer, with an initial learning rate of 0.005 and weight decay of 10^–5^. The XMR model was implemented using the PyTorch library and trained on a GPU server with an NVIDIA Tesla V100 32 GB GPU and an Intel Xeon Gold 6248 CPU.

## 3 Results

### 3.1 Heavyweight chemical embedding is critical to model performance

Following the “Construction of TNBC-specific XMR models” section, we constructed five TNBC-specific XMR models and compared them with two state-of-the-art approaches: DrugCell (Kuenzi et al., 2020) and ParsVNN (Huang X. et al., 2021). The architecture of DrugCell is depicted in Figure 1D, and it utilizes the VNN architecture as the genotypic embedder and a MLP as the chemical embedder. ParsVNN was built based on DrugCell and made the VNN in DrugCell more compact via pruning the VNN architecture, resulting in the architecture shown in Figure 1E.

**FIGURE 3:**
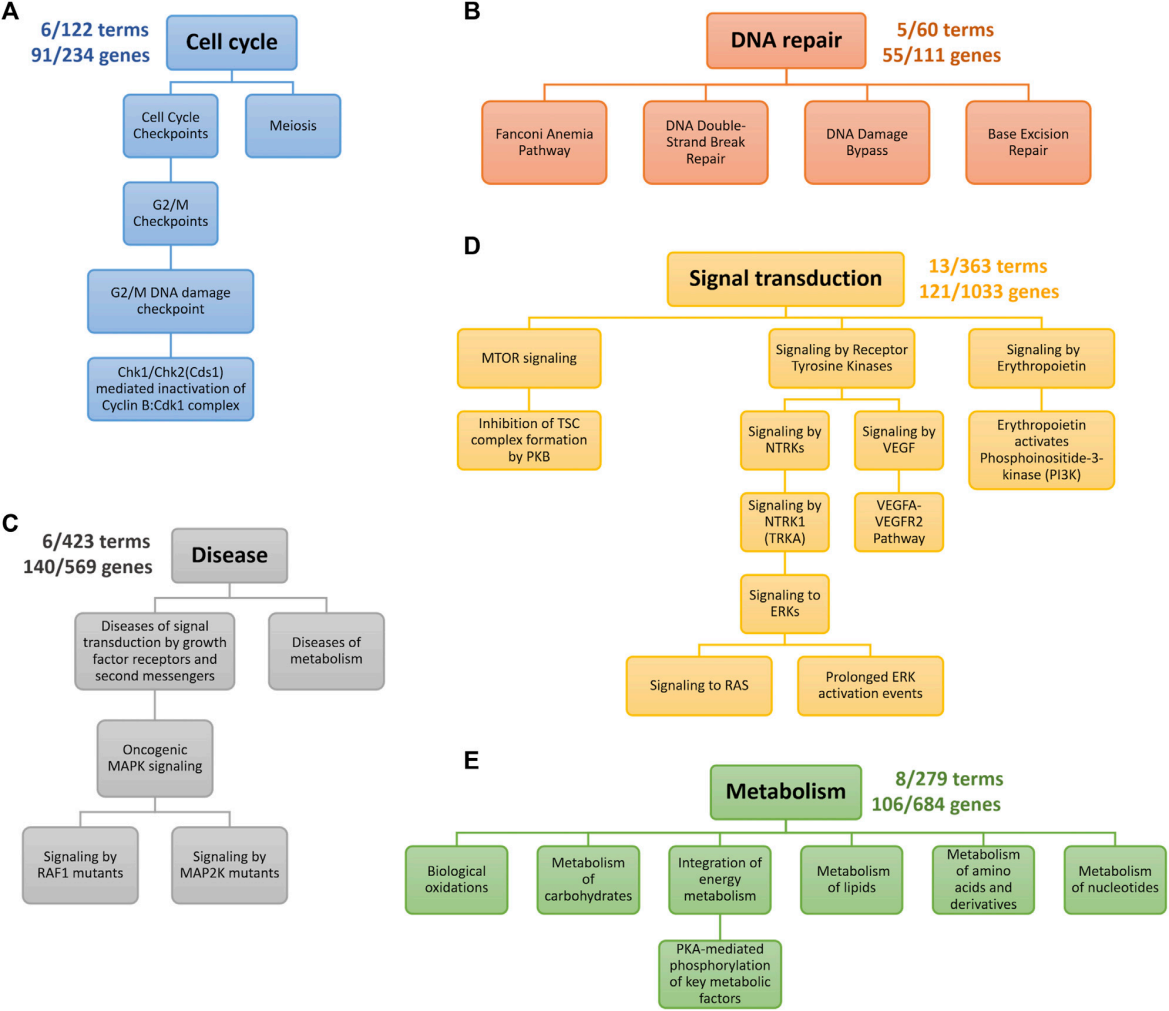
Genotypic pathways after pruning. Each color represents a distinct hierarchy. The numbers near the root term describe the number of terms and genes left. **(A)** the cell cycle pathway. **(B)** DNA repair pathway. **(C)** Pathways for Growth factor receptor- and metabolism-mediated diseases. **(D)** Pathways for signaling transduction. **(E)** Pathways for metabolism.

First, we observed that the TNBC-specific XMR models learned were highly compact, as illustrated in Figure 3. For instance, the XMR model constructed with the signal transduction biological network only had 13 terms and 121 genes remaining, which showed that approximately 96% of the terms and 88% of the genes were removed from the original VNN architecture. All the other TNBC-specific XMR models also had a limited number of terms and genes. This substantial reduction in the complexity of the genotypic embedder could enhance the generalizability of the VNN embedder.

**FIGURE 4:**
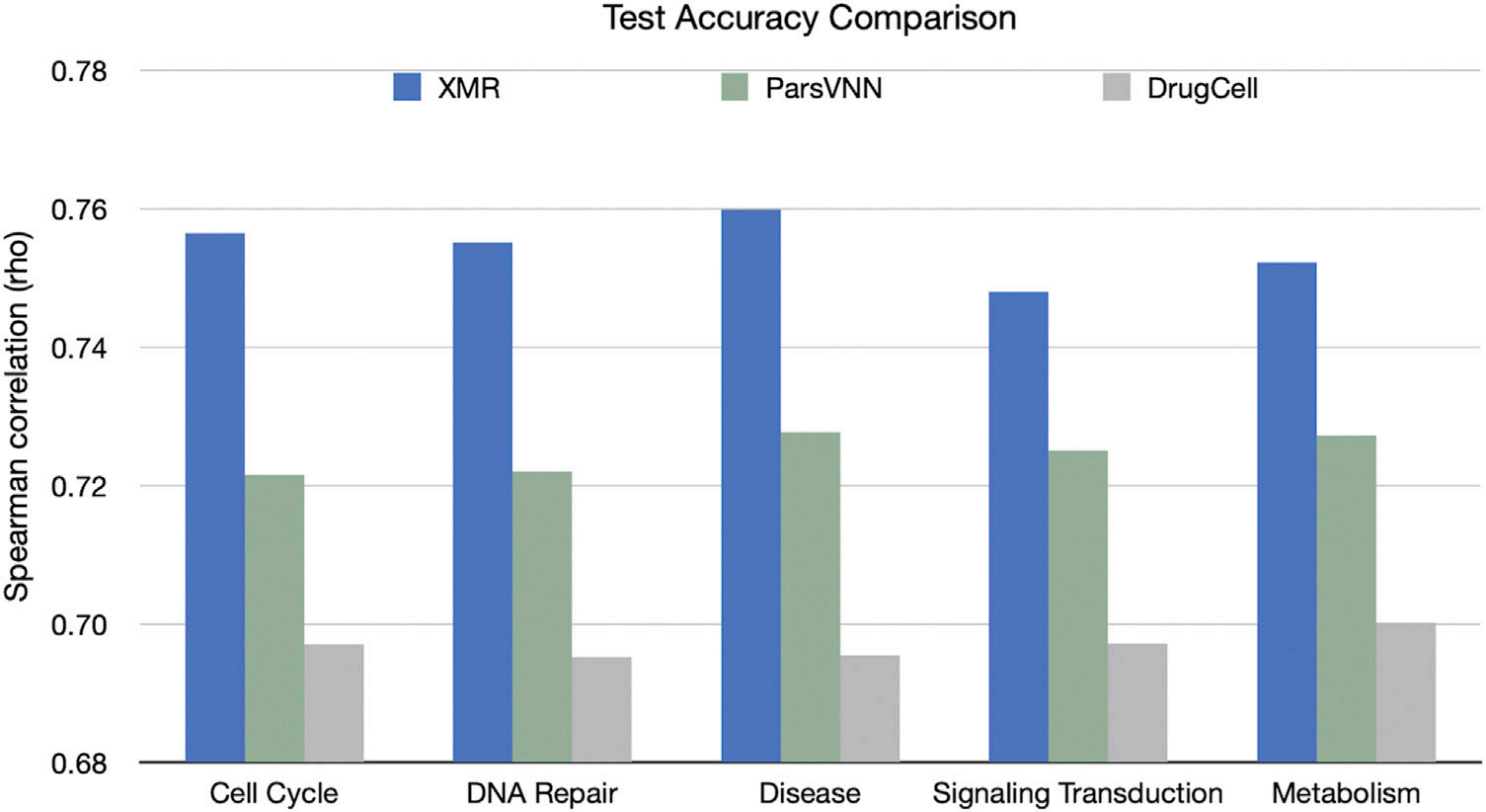
Comparison of XMR’s performance with other competing methods for predicting drug response across five separate pathways. Spearman’s correlation (rho) between predicted and observed drug responses was used as an evaluation criterion.

We also compared the performance of the TNBC-specific XMR models with the rival methods in terms of accuracy on the validation set (Figure 4). The results showed that XMR outperformed the other two methods with a minimum advantage of 2.3%. Furthermore, we used the method proposed by Diedenhofen and Musch (2015) to test the hypothesis that the correlation (shown in Figure 4) obtained by our model is not larger than the correlation (shown in Figure 4) obtained by a competing method. We found that in all the tests we performed, the *p*-value is smaller than 0.01, indicating that the correlation obtained by our model is significantly larger than the correlation obtained by the competing methods. These results further support the hypothesis discussed in Section 2.1. We can see that both a simplified genotypic embedder and a heavyweight chemical embedder contribute to the performance.

**FIGURE 5:**
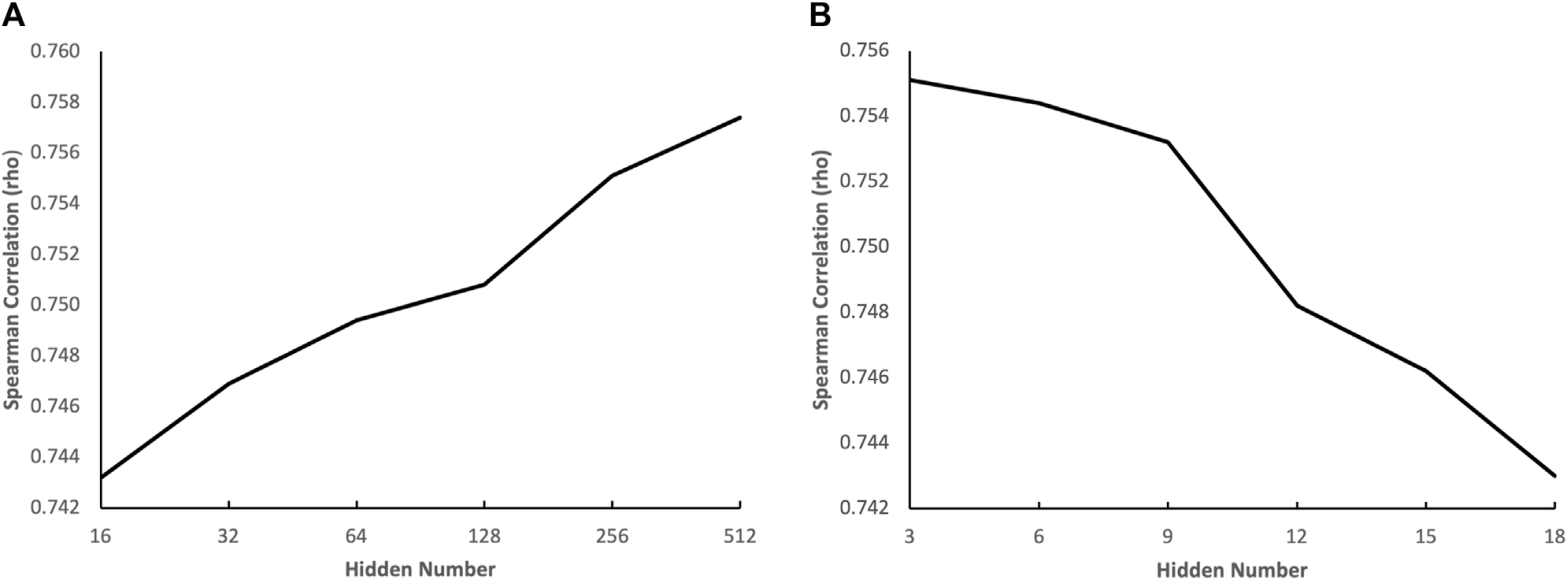
Impact of the number of hidden neurons on prediction accuracy. **(A)** the effect of the number of hidden neurons in the GNN on performance **(B)** the effect of the number of hidden neurons in the VNN on performance.

To gain a deeper understanding of how the complexity of the genotypic embedder and the chemical embedder influences performance, we conducted an ablation study to evaluate the impact of the number of hidden neurons of each term in both embedders on performance. The results are displayed in Figure 5. Figure 5A illustrates the effect of the number of hidden neurons in the GNN on performance, and we can see that the performance increases with the number of hidden neurons in the GNN. The performance continues to improve even when the number of hidden neurons is increased to 512, indicating that a heavyweight chemical embedder is necessary. Figure 5B shows the impact of the number of hidden neurons in the VNN on performance, and we can see that the performance deteriorates when the number of hidden neurons grows from three to six, indicating that the VNN is highly susceptible to overfitting. Therefore, a pruning method would greatly benefit the VNN, leading to a more lightweight genotypic embedder. These phenomena also align with our hypothesis.

### 3.2 Interpretations of genes and pathways

**FIGURE 6:**
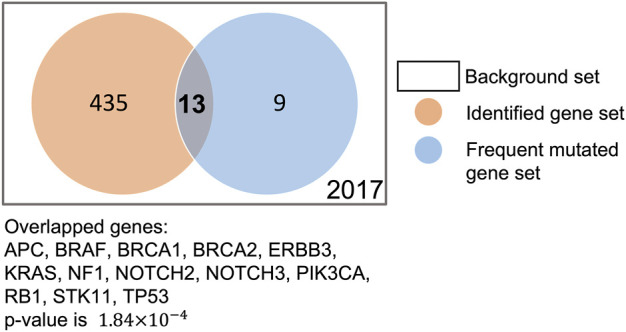
Comparison of genes identified by our model and frequently mutated genes in the literature.

To verify whether our XMR model can generate a reasonable gene-level explanation, we checked whether the commonly mutated genes in TNBC were preserved by our XMR model. We first extracted a series of commonly or frequently altered genes in TNBC (see Supplementary Material) from the literature on the PubMed database using the following search string: (triple-negative breast cancer OR TNBC) AND (commonly mutated genes OR highly frequently mutated genes). We found that our model identified 13 such genes out of 22 genes: *TP53*, *PIK3CA*, *BRCA1/2*, *RB1*, *NOTCH2/3*, *BRAF*, *ERBB3*, *APC*, *STK11*, *KRAS*, and *NF1* (Philipovskiy et al., 2020; Kudelova et al., 2022; Li et al., 2022). We further conducted Fisher’s exact test to evaluate their significance. The *p*-value of 1.84 × 10^−4^ indicated that these genes were not randomly selected (Figure 6). Although the model did not retain other reported frequently mutated genes [e.g., *PTEN*, *AKT1*, and *ATM*], it still remains reasonable, given that the mutation frequency of these genes greatly varies among multiple studies (Kudelova et al., 2022). Considering allowable uncertainty and variability in simulation, these findings supported the feasibility and plausibility of the modeling framework coupled with the pruning approach to identify likely critical genes.

We further checked the pathways identified by our XMR model. The retained pathways belonging to five categories of biological processes are graphically presented in Figure 3 and explained as follows. The genes corresponding to each term along individual pathways are provided in the Supplementary Material. Generally, our model found G2/M checkpoint, DNA repair-related, PI3K/mTOR signaling, RAS/RAF/MAPK/ERK signaling pathways, etc., which are enriched in different subtypes of TNBC [e.g., BL1, BL2, M, and LAR subtypes] (Lehmann et al., 2011; 2021).

1. Figure 3A indicates the pathway retained in the process of the cell cycle. The G2/M DNA damage checkpoint is likely sensitive to drug exposure based on our model. The loss of cell cycle checkpoints has been well-viewed as the hallmark of cancer (Löbrich and Jeggo, 2007). The remaining genes, including *TP53*, *RB1*, *BRCA1/2*, *STAG2*, and *TP53BP1*, are the common genes engaged in the cell cycle (Coussy et al., 2019; Kudelova et al., 2022). Another two genes (i.e., *RAD21* and *MDC1*) are likely associated with DNA damage, possibly implying a G2/M checkpoint loss as well (Lehmann et al., 2011). Despite limited research, gene functional analyses suggested the potential role of meiosis, given that the differentially expressed genes of TNBC were markedly enriched in the oocyte meiosis pathway (Cao et al., 2021).
2. A total of four pathways belonging to DNA repair were retained by our model (Figure 3B). DNA repair defects are thought to be more common than homologous recombination defects for breast cancer (Lee et al., 2019). Common genes relevant to this process (e.g., *TP53*, *RAD50*, and *POLE*) (Coussy et al., 2019; Lehmann et al., 2021; Kudelova et al., 2022) were also detected in this study. Our model identified four breast cancer susceptibility genes in the Fanconi anemia pathway: *FANCC*, *FANCD2*, and *FANCM* (Fang et al., 2020). *XRCC1*, a potentially critical gene associated with TNBC through both base excision repair and double-strand break repair pathways, was also detected by the model (Lee et al., 2019). Currently, limited data on the impacts of DNA damage bypass defects on TNBC development can be found.
3. Growth factor receptor- and metabolism-mediated diseases are shown in Figure 3C. Sufficient evidence has indicated that aberrant MAPK signaling is associated with TNBC occurrence (Jiang et al., 2020; Lehmann et al., 2021). Activating mutations in MAP2K and RAF1 (i.e., *MAP2K1*, *BRAF*, *KRAS*, and *NF1*) would dysregulate cellular proliferation, differentiation, and survival. These genes are common genes involved in the MAPK signaling pathway (Coussy et al., 2019). The metabolism-mediated process mainly captured two genes (i.e., *NOTCH2/3*) in NOTCH signaling. The dysfunction of NOTCH signaling could contribute to the development of many cancers (e.g., TNBC) (Bray, 2006; Lehmann et al., 2021).
4. The commonly accepted PI3K/mTOR pathway that affects TNBC, especially BL2 and LAR subtypes (Lehmann et al., 2021), is presented in Figure 3D. The common genes we identified in this pathway include *PIK3CA*, *PIK3R1*, *MTOR*, *STK11*, and *TSC1* (Coussy et al., 2019). Although the more straightforward pathway (i.e., PI3K/AKT signaling) was pruned, PI3K signaling activated by erythropoietin could sufficiently induce the proliferation of breast cancer cell lines (Tóthová et al., 2021). Additionally, the model also detected several mechanisms involved in the cascade by the phosphorylation of MAPK (i.e., RAS/ERK signaling) and VEGF signaling (Figure 3D). The aberrant activities of these pathways are potential factors for TNBC (Butti et al., 2018; Lehmann et al., 2021).
5. The metabolic dysregulation of TNBC may include remarkably altered amino acids, lipids, carbohydrates, nucleotides, and energy levels (Gong et al., 2021), which is also an emerging focus for cancer treatment (Sun et al., 2020). Similar molecular features of TNBC related to metabolism were observed by our model (Figure 3E). Targeting identified genes [e.g., *FASN* and *SLC7A5*] may provide a potential strategy to treat TNBC (Sun et al., 2020; Nachef et al., 2021), but the metabolism of TNBC still calls for further investigations due to limited studies.

### 3.3 Interpretations of drugs

In this section, we verified the top 10 drugs predicted to be effective by our XMR model. The top 10 drugs that would target TNBC were identified in consideration of both the predicted drug response and relatively adequate information on chemical–drug interactions provided by the PubChem database (Kim et al., 2022). The potentially effective drugs and corresponding information are summarized in Table 1. Specifically, the top 10 drugs derived from the synthesis of five major pathways (i.e., cell cycle, DNA repair, signaling transduction, metabolism, and diseases) are listed under “*Integrated results*,” followed by the drugs only identified by the individual pathways listed under “*Pathway-specific results*.” It is worth noting that the top drugs were screened and selected based on the (cell line and drug) pairs that were not presented in the entire dataset, thereby reflecting the prediction capability of our model. In addition, platinum salts, a classical group of alkylating agents applied in neoadjuvant chemotherapy for TNBC (Nedeljković and Damjanović, 2019), were not included in the drug repository we utilized in this study, since we aimed to gain a better understanding of the efficacy of relatively novel drugs.

**TABLE 1:** Integrated and pathway-specific top 10 drugs identified by XMR and their corresponding descriptions.

Drug	Case no.	Predicted AUC value	Category	Clinical study
*Integrated results*				
Leptomycin B (LMB)	87081-35-4	0.248	XPO1 inhibitor	NA
Dinaciclib	779353-01-4	0.284	CDK1/2/5/9 inhibitor	NCT01624441
				NCT01676753
AZD7762	860352-01-8	0.323	Checkpoint kinase inhibitor	NA
Ouabain	630-60-4	0.376	Plasma membrane *Na* ^(+)^/*K* ^(+)^-ATPase inhibition	NA
Panobinostat	404950-80-7	0.378	Histone deacetylase inhibitor	NCT02890069
				NCT01105312
BI 2536	755038-02-9	0.436	Polo-like kinase 1 inhibitor	NCT00526149 (breast cancer (BC))
Homoharringtonine	26833-87-4	0.447	Mcl-1 protein synthesis inhibitor	NA
TW-37	877877-35-5	0.456	Bcl-2 inhibitor	NA
Cytarabine	147-94-4	0.460	DNA polymerase inhibitor	NCT01645839 (BC)
				NCT00992602 (BC)
GSK461364	929095-18-1	0.465	Polo-like kinase 1 inhibitor	NA
*Pathway*-*specific results*				
Vincristine	57-22-7	-	Target microtubules and mitotic tubulin	NCT02299999 (BC)
Thapsigargin	67526-95-8	-	Sarco-endoplasmic reticulum *Ca* ^2+^-ATPase modulator	NA
Bleomycin	11056-06-7	-	DNA damage	NCT00744653 (BC) terminated

Generally, the five pathways identified similar drugs. Four drugs have been studied in recent clinical trials to explore their potentials to treat TNBC or breast cancer (BC) with leptomeningeal metastases (i.e., dinaciclib, panobinostat, BI 2536, and cytarabine). Although the majority of clinical trials utilize combination therapies, these drugs may play a role in a synergistic way. The other four drugs are still under *in vitro* or *in vivo* investigations of TNBC treatment (i.e., AZD7762, ouabain, homoharringtonine, and GSK461364) (Giordano et al., 2019; Yakhni et al., 2019; Du et al., 2021; Zhu et al., 2021; Plett et al., 2022). Interestingly, leptomycin B (LMB) was considered unsafe in previous clinical trials due to its adequate adverse effect and thus not approved for use (Wang and Liu, 2019), while it is the most effective drug identified by our model. As an alternative to LMB, selinexor exhibits manageable side effects and processes a similar mechanism. It shows the potential to treat TNBC based on several preclinical studies (Cheng et al., 2014; Arango et al., 2017) and clinical trials (e.g., NCT05035745, NCT02402764, and NCT02419495). If selinexor was included in our drug repository, it would probably be identified instead of LMB. For pathway-specific drugs, BC-related clinical trials using vincristine and bleomycin can be found, but bleomycin is usually applied to evaluate the effects of electrochemotherapy (Radica et al., 2020).

From the point of mechanism-based view, the majority of the top agents target the cell cycle process, DNA replication, and DNA repair (e.g., LMB, dinaciclib, AZD7762, panobinostat, BI 2536, homoharringtonine, TW-37, cytarabine, and GSK461364), which are in line with part of the characteristics of BL1 and BL2 subtypes (Lehmann et al., 2011; Lehmann et al., 2021). Although there are limited studies on the effects of TW-37 on TNBC (Table 1), it does not necessarily mean that TW-37 is unpromising. Since myeloid cell leukemia-1 (Mcl-1) is a critical factor for the survival and motility of TNBC cells (Goodwin et al., 2015), TW-37 likely plays a key role in TNBC treatment. Two drugs are characterized by ion channel transport (i.e., ouabain and thapsigargin), which may be related to critical signaling pathways for TNBC. For example, it was shown that overexpression of the NOTCH signaling pathway (e.g., NOTCH 1) would induce proliferation and tumorigenesis of TNBC (Nedeljković and Damjanović, 2019), especially for BL1 and M subtypes. As a sarco-endoplasmic reticulum *Ca* ^2+^-ATPase (SERCA) modulator, thapsigargin may inhibit oncogenic NOTCH 1 signaling (Pagliaro et al., 2021), thereby possibly suppressing tumor growth.

## 4 Discussion

Predicting drug response in cancer patients can be difficult due to the genetic diversity among them. The end-to-end training of such a model requires balancing the representation of two types of data fed into the model while also serving its prediction purpose, i.e., accurately projecting the relationship between the two modalities. There has been limited exploration in this area. For example, DrugCell used the VNN instead of the usual neural network to fully incorporate biological processes at molecular and cellular levels (Kuenzi et al., 2020). Another effort leveraged ParsVNN to select important terms and edges of those biological architectures to improve the performance and explanation (Huang X. et al., 2021). However, ParsVNN still used the simple MLP to extract features from the Morgan fingerprint of drugs similar to DrugCell (Kuenzi et al., 2020). The information on drugs’ molecular structures is not fully used in those models. Meanwhile, there have been many studies in the field of vision-and-language processing (VLP) tasks. Those insights and results were gained from developing multimodal models for VLP, which can be effectively applied to drug response prediction. In this paper, we have developed XMR, which follows a similar structure to ParsVNN for the gene information (Huang X. et al., 2021) but uses the GNN to extract the information between neighbor atoms from the molecular structure of a certain chemical (Tsubaki et al., 2019).

We chose drug response data in TNBC cell lines from GDSC (Yang et al., 2012) and CTRP (Seashore-Ludlow et al., 2015). Selecting this disease as the case in this study is primarily because TNBC treatment (e.g., classical regimens) still remains challenging compared to other types of breast cancer, given the lack of specific hormone receptors, common driver mutations (Rajput et al., 2016; Zhu et al., 2022), and high heterogeneity and resistance (Nedeljković and Damjanović, 2019). This brings us to seek novel personalized approaches to treating TNBC. In this study, we compared our model with the existing methods under five biological pathways obtained from the Reactome Pathway Database (Fabregat et al., 2018). It indicates that XMR outperforms both DrugCell and ParsVNN in terms of test accuracy (Figure 4). Moreover, the results derived from the XMR model can be explained and verified at levels of genes, pathways, and drugs (Figure 3).

Overall, 13 commonly or frequently mutated genes related to TNBC were retained by our model. We also identified commonly accepted pathways (e.g., cell cycle, DNA repair, PI3K/mTOR, and MAPK signaling) and promising pathways associated with metabolic reprogramming. Additionally, several novel drugs tested under clinical trials/cell experiments/animal studies were selected (Table 1). For the purpose of selecting new drugs, we ensured that the test dataset contained as many novel (cell line and drug) pairs as possible. In other words, the (cell line and drug) pairs applied for drug screening have ruled out all combinations shown in both training and validation datasets from previous experiments. This way, the commonly used agents for TNBC, such as DNA-damaging agents (e.g., doxorubicin and cyclophosphamide) and mitotic inhibitors (e.g., docetaxel) (Powell et al., 2020), and recently discovered agents, such as poly(ADP-ribose) polymerase (PARP) inhibitors (olaparib, veliparib, and rucaparib), were ultimately not considered in this study. For example, in the test dataset, only four combinations among a total of 1,864 (cell line and drug) pairs were relevant to docetaxel, not to mention doxorubicin which had no pairs to be tested.

Nevertheless, there are some underlying limitations that would be addressed by future efforts. First, our model contains three components: a genotypic embedder, a chemical embedder, and a modality interaction block. In this study, we focused on balancing the complexity of the first two embedders while maintaining the design of the modality interaction block simple. However, the modality interaction block has been recognized as an essential element in VLP tasks, as demonstrated in studies such as ViLBERT (Lu et al., 2019), UNITER (Chen et al., 2020), and ViLT (Kim et al., 2021). It allows us to improve the interaction in the future by employing a multi-headed self-attention layer to extract more comprehensive features in the interaction between the two modalities. Second, GDSC provides drug response data with multi-drugs (Huang S. et al., 2021), while the current model only considers the effects of a single drug. The model could be further refined by synthesizing disparate types of drugs (e.g., classical regimens and immunotherapies) and by delving into their synergistic effects to better facilitate TNBC treatment.

Although our model showed the ability to provide biologically reasonable interpretations, most drugs exhibit mechanisms that are associated with cell cycle and DNA repair. Apart from the property of our test dataset itself (e.g., perhaps uneven distributions of cell-line data), the pathways utilized in this study may be another contributor. Currently, our model is characterized by five pathways, including cell cycle, DNA repair, diseases, signaling transduction, and metabolism, listed in the Reactome Pathway Database (Fabregat et al., 2018). However, other pathways, such as the immune system, developmental biology, and hemostasis, have also been reported as having potential linkages with TNBC development, especially the specific subtypes (Lehmann et al., 2011; Lehmann et al., 2021). These pathways may inform the VNN structure and lead to more comprehensive results of drug discovery when incorporated into the model. Additionally, distinct databases of biological processes (e.g., Gene Ontology (GO), Kyoto Encyclopedia of Genes and Genomes (KEGG), and Reactome) may differ in their ultimate findings, due to different annotations and genes they cover. Although the comparison of the model performance based on these databases is beyond the scope of our study, it would be an invaluable factor in refining the interpretability of our model.

## Data Availability

The original contributions presented in the study are included in the article/Supplementary Material; further inquiries can be directed to the corresponding author. The source code of XMR and the datasets are available at https://github.com/zwa2/XMR.
