# Supplementary material for: XMR: an explainable multimodal neural network for drug response prediction
Source: Front Bioinform. 2023 Aug 2;3:1164482. doi: 10.3389/fbinf.2023.1164482 (PMC10433751; doi:10.3389/fbinf.2023.1164482)
Supplement: Supplementary file 1 [file DataSheet1.PDF]

Supplementary Material

1 GENES AND TERMS REMAINED IN OUR MODEL

All terms and genes are from Reactome(Fabregat et al., 2018).

1.1 Cell cycle

R-HSA-1640170 (Cell Cycle)
AKAP9, BARD1, BRCA1, CCNB2, CDC14A, CENPJ, CENPT, CHTF18, DCTN1, DKC1, DMC1, DNA2, ESCO1, GSK3B, H2BC9, INCENP, JAK2, KIF18A, LBR, LIN9, MASTL, MCM6, MCPH1, MSH4, NDC1, NSD2, NUMA1, NUP155, NUP205, NUP43, ODF2, PIAS4, POLE, POM121, PPP1R12A, PRIM1, PSMB10, PSMC3, PSMD7, PSME4, PTK6, RAB8A, RAD17, RAD21, RAD50, RANBP2, RB1, RFC2, RMI2, RRM2, RUVBL1, SGO2, SIRT2, SMC4, STAG2, SUN2, TEX15, TOP2A, TUBA3E, UBE2D1, YWHAZ

R-HSA-1500620 (Meiosis)
BLM, BRCA2, DMC1, FKBP6, H2AC14, MLH3, MSH4, PRDM9, RAD21, RAD50, SMC3, TEX15, UBE2I

R-HSA-69481 (G2/M Checkpoints)
BARD1, BRCA1, CCNB2, CDC45, DNA2, MCM5, MCM6, MDC1, NSD2, ORC4, PIAS4, PSMB10, PSMC3, PSMD7, PSMD8, PSME4, RMI2, TOP3A, TP53, TP53BP1, YWHAZ

R-HSA-69473 (G2/M DNA damage checkpoint)
BARD1, BLM, BRCA1, BRIP1, DNA2, H2BC17, H2BC9, HERC2, KAT5, MDC1, NSD2, RAD17, RAD50, RFC2, RMI2, RNF8, TOP3A, TP53, TP53BP1, YWHAZ

R-HSA-69620 (Cell Cycle Checkpoints)
BRCA1, CCNB2, CENPE, CENPO, CENPT, CENPU, CLASP2, DNA2, DYNC1LI2, INCENP, ITGB3BP, KNL1, MCM6, NUP107, PSMC3, RAD17, RANBP2, RMI2, SEC13, UBE2D1, YWHAZ

R-HSA-75035(Chk1/Chk2(Cds1) mediated inactivation of Cyclin B:Cdk1 complex)
YWHAZ

1.2 DNA Repair

R-HSA-73894 (DNA Repair)
BAZ1B, CDK2, COPS2, COPS6, DNA2, ERCC3, ERCC5, FANCC, FANCI, H2AC14, MSH3, MSH6, NTHL1, PAXIP1, POLE, POLH, RMI2, TP53, TP53BP1, UBE2B, UBE2I, USP45, UVSSA, WDR48, XAB2

R-HSA-73884 (Base Excision Repair)
APEX1, H2AC14, H2BC17, LIG3, NEIL1, NTHL1, PARG, PNKP, POLD1, RFC2, XRCC1

R-HSA-73893 (DNA Damage Bypass)
DTL, MAD2L2, POLH, REV1, RFC2, UBA7, WDR48

R-HSA-5693532 (DNA Double-Strand Break Repair)
BAP1, BARD1, BAZ1B, EME1, H2BC17, PAXIP1, PIAS4, POLH, PPP4R2, PRKDC, RAD17, RAD50, RAD51C, RFC2, TP53, TP53BP1, UBE2I, XRCC1

R-HSA-6783310 (Fanconi Anemia Pathway)
EME1, EME2, ERCC4, FAAP100, FANCA, FANCC, FANCD2, FANCF, FANCG, FANCI, FANCM, WDR48

1.3 Disease

R-HSA-1643685 (Disease)
ACY1, ADAMTS12, ADAMTS20, ADAMTSL3, AGGF1, AGTRAP, APC, APOA1, ARID4B, CSPG5, DUSP10, EEF2, ERBB3, ERBB4, ERLIN2, EZH2, FADD, FOXO1, FURIN, GHRH, HDAC9, HNRNPK, HRH2, IKBKG, IRS2, JAK2, KAT2B, KPNA7, LRP6, LUM, MAP2K1, MAPKAP1, MC5R, MGAT5, MUC16, NEIL3, NELFB, NOTCH3, NOX4, NPSR1, NR3C1, NUP43, PARP14, PC, PDGFRA, PGK1, POM121, PTGDR, PTGER4, RCOR1, RPL26L1, RPL3L, SAP18, SEL1L, SI, SLC22A12, SLC2A2, SLCO1B3, SPON1, STRN, STT3A, SUPT16H, TAAR2, TBXAS1, TPST2, TXNIP, TYK2, UBE2D1, VAV3

R-HSA-5668914 (Diseases of metabolism)
ADAMTS20, ADAMTSL3, ADAMTSL4, ADAMTSL5, BTD, CHSY1, CSPG5, EXT2, FMOD, GAA, GALK1, GALNT12, GPC1, GPC2, KERA, LUM, MAOA, MAT1A, MCCC1, MUC6, NAGLU, NHLRC1, NOTCH2, NOTCH3, PC, PMM2, RFT1, SEMA5B, SLC34A2, SPON1, TBXAS1

R-HSA-6802957 (Oncogenic MAPK signaling)
AGTRAP, ATG7, CNKSR1, DUSP10, DUSP16, DUSP8, FAM114A2, FXR1, IQGAP1, JAK2, MAP2K1, MAP3K11, MARK3, NF1, SND1, TLN1, TRAK1, TRIM24

R-HSA-9656223 (Signaling by RAF1 mutants)
ARAF, BRAF, CNKSR1, ITGB3, JAK2, KRAS, MAP2K1, MARK3, RAF1, VWF

R-HSA-5663202 (Diseases of signal transduction by growth factor receptors and second messengers)
AGGF1, AGTRAP, ARAF, AXIN1, BCR, CUX1, DUSP10, DUSP16, EP300, ERBB3, ERBB4, ERLIN2, ESR2, FOXO3, FZD6, FZD8, HDAC8, IQGAP1, ITGA2B, JAK2, LRP5, LRP6, MAML1, MAP2K1, MPRIP, NCOR2, NCSTN, PDGFA, PDGFB, PDGFRA, PDGFRB, PORCN, PSMB10, PSMD7, PSMD8, PSME4, RAC1, SEL1L, SNW1, STRN, TGFB1, TRAT1, TRIM24, TRIP11, VWF

R-HSA-9652169 (Signaling by MAP2K mutants)
MAP2K1

1.4 Signal Transduction

R-HSA-162582 (Signal Transduction)
ABCA1, ABCG5, ADAM12, AGO3, ARHGAP20, ARHGEF10L, ATP6V0B, AXIN1, CENPT, CIT, CMKLR1, CXXC5, DAAM1, DOCK1, DUSP10, EZH2, FASN, FGD1, GABRQ, GOLGA7, HRH2, HRH3, IKBKG, IRS2, JAK2, KDR, KLC2, KLC4, LAMA5, LAMB3, LRP6, MC5R, MTNR1B, NBEA, NCOA1, NGF, NOG, NOTCH3, NPSR1, OR10X1, OR2M2, OR2S2, OR2T2, OR2T8, OR4B1, OR4D10, OR4D5, OR4D6, OR52B6, OR52N2, OR5B2, PDE3A, PDE4B, PDGFRA, PLPPR1, PRKACB, PRKAG2RAPGEF4RCOR1RGS3RPS6KA5, SHC2, SOX7, SPTBN2, SSTR4, STAT6, STRN, TAAR2, TAS2R19, TAS2R30, TAS2R5, TSC1, UBE2D1, WASF2, WNT11, YAP1

R-HSA-187687 (Signalling to ERKs)
NTRK1, SHC2

R-HSA-169893 (Prolonged ERK activation events)
BRAF, CRK, MAP2K1, NTRK1

R-HSA-167044 (Signalling to RAS)
NGF, NTRK1, SHC2

R-HSA-9027276 (Erythropoietin activates Phosphoinositide-3-kinase (PI3K))
GAB1, IRS2, JAK2, PIK3CA, PIK3CG, PIK3R1

R-HSA-4420097 (VEGFA-VEGFR2 Pathway)
ELMO1, MAPKAP1, PIK3CA, PTK2, SHC2, VAV3, WASF2

R-HSA-194138 (Signaling by VEGF)
ELMO1, MAPKAP1, VAV3, WASF2

R-HSA-166520 (Signaling by NTRKs)
ATF2, BRAF, DOCK3, ID4, IRS2, MAPK13, MEF2A, NELFB, NTRK2, PIK3R1RPS6KA5, SH3GL2, SHC2, TF, TPH1

R-HSA-9006934 (Signaling by Receptor Tyrosine Kinases)
AXL, COL6A5, CTNNA1, DNM1, EP300, GABRB2, GABRB3, GABRG3, ITGB3, MAPKAP1, NCSTN, PDGFRA, PIK3R4, PLG, PTK6, PTPRJ, TIAM1

R-HSA-165181 (Inhibition of TSC complex formation by PKB)
TSC1

R-HSA-9006335 (Signaling by Erythropoietin)
GAB1, JAK2, PIK3CA, PIK3R1

R-HSA-165159 (MTOR signalling)
AKT1S1, EIF4G1, MTOR, PRKAG2, STK11, TSC1

R-HSA-187037 (Signaling by NTRK1 (TRKA))
ELK1, EP300, IRS2, MEF2A, NTRK1, NTRK2, PIK3R2RHOARPS6KA5, SGK1, SHC2, TPH1

1.5 Metabolism

R-HSA-1430728 (Metabolism)
AGXT, ALDH1B1, AOX1, ARSD, CHSY1, CPNE6, CREBBP, CYP2D6, DMGDH, ELOVL4, ENPP7, FDFT1, FITM2, GALK1, GART, GATM, HADHB, HAO1, HGD, MARS1, MLXIPL, MT-ND5, MT-ND6, MTF1, NCOA1, NT5C1A, OCA2, OSBPL6, OXCT2, PC, PGP, PI4KB, PIP5K1B, PLA2G4A, PLCB2, PLCH2, POLD1, POM121, PRKAG2, PTGS2, PTPMT1, PYCR1, RETSAT, RPL26L1, SEC24B, SLC7A5, SLC9A1, SLCO1B3, SULT1E1, TPH1, UGT2B4

R-HSA-71387 (Metabolism of carbohydrates)
B3GALT4, B4GALNT2, CHSY1, FMOD, GALK1, NDST4

R-HSA-211859 (Biological oxidations)
ACSM2A, ACY1, ALDH1B1, CYP1A1, CYP2D6, FMO3, GGT6, GSTZ1, NNMT, NR1H4, SULT1A2, UGT2A3, UGT2B4

R-HSA-163685 (Integration of energy metabolism)
ACACB, ADCY8, CACNA1A, CACNA1C, CACNA1E, CD36, FASN, FFAR1, IQGAP1, ITPR1, KCNC2, MLXIPL, PLCB1, PLCB2, PLCB3, PRKAG2, RAPGEF3, SNAP25, STXBP1

R-HSA-556833 (Metabolism of lipids)
ACOT12, ACOT2, ARSD, BCHE, CPNE7, ETNK1, FDFT1, OSBPL6, OXCT2, PON1, PTPMT1, SEC24B, SEC24C, SLC27A1, TPTE2

R-HSA-163358 (PKA-mediated phosphorylation of key metabolic factors)
MLXIPL

R-HSA-71291 (Metabolism of amino acids and derivatives)
DMGDH, GATM, HAAO, HAO1, PSMB10, PSMD7, PSME4, PYCR1, QARS1, RPS25, SLC5A5, SLC7A5, SRM

R-HSA-15869 (Metabolism of nucleotides)
DTYMK, ENTPD2, ENTPD7, GART, GMPR2, NT5C1A, NT5M, NUDT18, RRM2, SAMHD1

2 COMMONLY OR FREQUENTLY ALTERED GENES OF TNBC

There are 2017 genes used in total five pathways for the construction of our modeling framework, and 448 unique genes remained after pruning. We extracted 41 commonly or frequently altered genes in TNBC from litterateurs on PubMed database (Kudelova et al., 2022; Li et al., 2022; Philipovskiy et al., 2020). Only 22 of them are in the set of background genes (2017 genes): APC, BRAF, BRCA1, BRCA2, COL6A3, EGFR, ERBB2, ERBB3, FGFR1, JAK3, KRAS, NF1, NOTCH1, NOTCH2, NOTCH3, PIK3CA, PTEN, RB1, STK11, SYNE1, SYNE2, TP53.

REFERENCES

Fabregat, A., Jupe, S., Matthews, L., Sidiropoulos, K., Gillespie, M., Garapati, P., et al. (2018). The reactome pathway knowledgebase. *Nucleic acids research* 46, D649–D655

Kudelova, E., Smolar, M., Holubekova, V., Hornakova, A., Dvorska, D., Lucansky, V., et al. (2022). Genetic heterogeneity, tumor microenvironment and immunotherapy in triple-negative breast cancer. *International Journal of Molecular Sciences* 23, 14937

Li, Y.-Z., Chen, B., Lin, X.-Y., Zhang, G.-C., Lai, J.-G., Li, C., et al. (2022). Clinicopathologic and genomic features in triple-negative breast cancer between special and no-special morphologic pattern. *Frontiers in Oncology* , 1124

Philipovskiy, A., Dwivedi, A. K., Gamez, R., McCallum, R., Mukherjee, D., Nahleh, Z., et al. (2020). Association between tumor mutation profile and clinical outcomes among hispanic latina women with triple-negative breast cancer. *PLoS One* 15, e0238262
